# Enhancing circulatory myokines and extracellular vesicle uptake with targeted exercise in patients with prostate cancer (the MYEX trial): a single-group crossover study

**DOI:** 10.1186/s12885-024-12530-0

**Published:** 2024-07-01

**Authors:** Jin-Soo Kim, Dennis R. Taaffe, Daniel A. Galvão, Timothy D. Clay, Andrew D. Redfern, Elin S. Gray, Robert U. Newton

**Affiliations:** 1 Exercise Medicine Research Institute, Edith Cowan University, Joondalup, WA Australia; 2 School of Medical and Health Sciences, Edith Cowan University, Joondalup, WA Australia; 3 Department of Medical Oncology, St John of God Subiaco Hospital, Perth, WA Australia; 4 Department of Medical Oncology, Fiona Stanley Hospital, Murdoch, WA Australia; 5 School of Medicine and Pharmacology, University of Western Australia, Perth, WA Australia; 6 Centre for Precision Health, Edith Cowan University, Joondalup, WA Australia; 7 School of Human Movement and Nutrition Sciences, University of Queensland, St Lucia, QLD Australia

**Keywords:** Prostate cancer, Exercise, Myokines, Extracellular vesicles

## Abstract

**Introduction:**

Physical activity is associated with improved disease progression and cancer-specific survival in patients with prostate cancer (PCa). However, the mechanisms underlying these associations remain unclear, while the relative impact of exercise modes is unknown. This study aims to examine the differential impact of exercise mode on tumour-suppressive skeletal muscle-associated systemic molecules as well as their delivery mechanism. This study will compare the effects of the two main exercise modes, aerobic and resistance, on (1) circulatory myokine levels, (2) skeletal muscle-induced extracellular vesicle abundance and cargo contents, and (3) uptake of extracellular vesicles (EVs) in PCa cells in patients with localised or advanced PCa.

**Methods:**

A single-group cross-over design will be used for patients at opposite ends of the disease spectrum. A total of 32 patients (localised PCa, *n* = 16; metastatic castrate-resistant PCa, *n* = 16) will be recruited while capitalising on two ongoing studies. Ethics amendment has been approved for two ongoing trials to share data, implement the acute exercise sessions, and collect additional blood samples from patients. The patients will undertake two exercise sessions (aerobic only and resistance only) in random order one week apart. Blood will be collected before, after, and 30 min post-exercise. Circulating/EV-contained myokine levels (irisin, IL-6, IL-15, FGF-21, and SPARC) and plasma skeletal muscle-induced EVs will be measured using ELISA and flow cytometry. PCa cell line growth with or without collected plasma will be examined using PCa cell lines (LNCaP, DU-145, and PC-3), while evaluating cellular uptake of EVs. Ethics amendments have been approved for two capitalising studies to share data, implement acute exercise sessions and collect additional samples from the patients.

**Discussion:**

If findings show a differential impact of exercise mode on the establishment of an anti-cancer systemic environment, this will provide fundamental knowledge for developing targeted exercise prescriptions for patients with PCa across different disease stages. Findings will be reported in peer-reviewed publications and scientific conferences, in addition to working with national support groups to translate findings for the broader community.

**Trial registration:**

The registration for the two capitalising studies are NCT02730338 and ACTRN12618000225213.

## Introduction

Exercise is well demonstrated to be effective in improving health-related outcomes in patients with cancer [1]. Specifically in patients with prostate cancer (PCa), exercise improves patient-reported outcomes and a range of treatment-related adverse effects [2, 3]. In observational studies 61% and 57% reductions in PCa-specific mortality [4] and disease progression [5], respectively, have been reported in patients who participated in physical activity for more than 3 h per week. Although these studies demonstrate the value of exercise therapy for patients with PCa, the causality and mechanisms behind these associations remains unclear [6]. A better understanding of the beneficial influence of exercise on tumour biology would permit the implementation of more targeted and effective exercise medicine in cancer management. A priority is to examine any differential effects of the two primary exercise modes: aerobic and resistance.

While multiple mechanistic hypotheses are proposed to explain how exercise influences tumour biology, the potential tumour-suppressive role of exercise-induced systemic changes have been demonstrated in multiple studies [6, 7]. Kang and colleagues in 2021 reported a significant reduction in PCa cell growth when PCa cells were directly exposed to serum collected from patients undergoing active surveillance after 12 weeks of aerobic exercise [8]. Similarly, we reported a significant growth reduction of PCa cells when cells were directly exposed to serum obtained after 3 months and 6 months of mixed-mode exercise (aerobic and resistance) in patients with localised PCa undertaking active treatment (androgen deprivation therapy [ADT]) [9] and patients with metastatic castrate-resistant prostate cancer (mCRPC) [10], while altering circulatory levels of skeletal muscle-produced cytokines called myokines [9, 10]. Further, our follow-up study examining acute exercise response showed transient elevation of circulating myokines (oncostatin M [OSM], secreted protein acidic and rich in cysteine [SPARC], IL[interleukin]-6, IL-15) after a single high-intensity aerobic exercise session in already exercise trained-patients with mCRPC accompanied by a significant reduction of PCa cell growth when cultured cells were exposed to exercise-conditioned serum [11]. These findings suggest that exercise in patients with PCa at various stages has the capacity to alter circulatory myokines, and that these alterations in circulatory myokines influence cancer cell growth in vitro experiments [7, 9–11]. However, while our previous studies provided fundamental information about the anti-tumour effect of exercise – circulatory myokine alterations, the differential impact of exercise features (frequency, intensity, time, and type [FITT]) on tumour suppressive skeletal muscle-induced molecules [6, 7] and how skeletal muscle-induced anti-tumour factors are delivered remains to be elucidated [12].

**Fig. 1:**
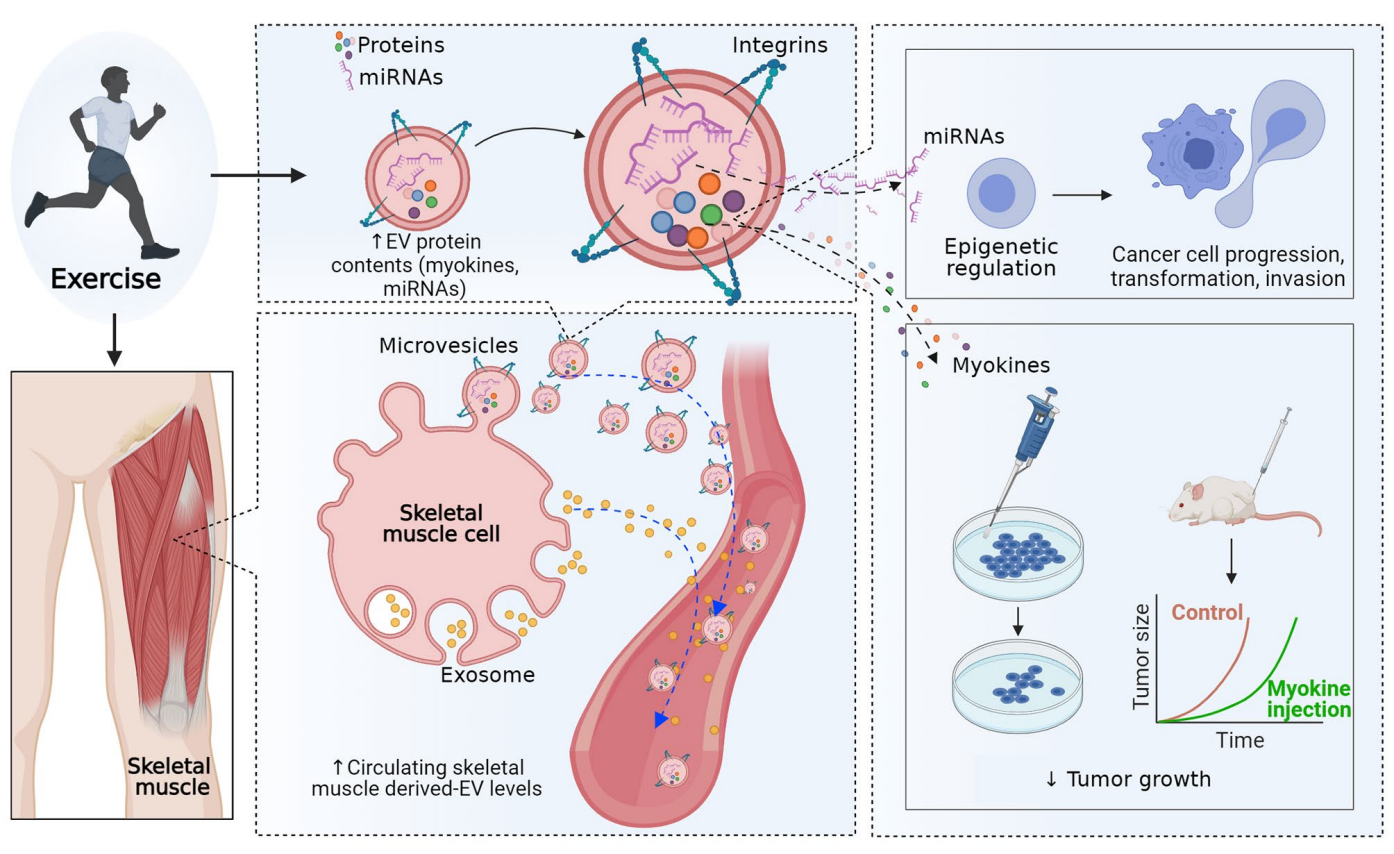
Effect of exercise on extracellular vesicles. Reprinted with permission from Zhang et al. ^12^ Exercise may increase the secretion of extracellular vesicles into the circulatory system and alter the concentration of proteins and microRNAs (miRNAs) in skeletal muscle-derived extracellular vesicles. MicroRNAs in skeletal muscle-derived extracellular vesicles may induce epigenetic regulation in prostate cancer cells and reduce cell progression, transformation, and invasion. In addition, skeletal muscle-derived extracellular vesicles containing proteins, including myokines, have a direct tumour-suppressive effect

To expand our understanding of the underlying mechanisms by which exercise influences tumour biology, we recently reviewed the role of skeletal muscle-induced extracellular vesicles (SMEVs) [12]. As an endocrine organ, skeletal muscle releases anti-cancer myokines both in cell-free/soluble forms as well as within SMEVs in response to exercise stimulation [12]. The tumour-suppressive effect of SMEV cargos (e.g., myokines) has been demonstrated in various cancer cells, including PCa [6, 12] (Fig. 1). In addition, the potential involvement of skeletal muscle-induced myokines in facilitating SMEV uptake in PCa cells has been suggested with the recent discovery of myokine-integrin interaction [13] The interaction between α_v_ family integrin and the myokine irisin (a cleaved form of Fibronectin type III domain containing 5 [FNDC5]) has been demonstrated in osteoblasts and adipocytes, with this interaction to increasing uptake of extracellular vesicles in these cells [13]. As α_v_ family integrins, such as integrin α_v_β_3_ and α_v_β_6_, are known to be abundantly expressed in PCa cells [14, 15], we hypothesised that exercise-induced elevation of circulatory myokines within SMEVs might be one of the mechanisms for delivering anti-oncogenic factors to tumour cells through this integrin interaction [12]. In addition, although a substantial increase of circulatory SMEVs was previously demonstrated after a single bout of exercise in normal murine models [16, 17] and healthy humans [16, 18–20] there is no existent knowledge on the role of exercise in circulatory extracellular vesicles in patients with cancer, including PCa.

The aim of the MYEX trial is to determine if exercise mode influences circulating myokine levels and the degree of uptake of extracellular vesicles into PCa cells specifically by implementing aerobic-only and resistance-only exercise protocols for patients with localised and advanced disease. Further, as hypothesised in our previous review [12], we aim to examine whether exercise-induced circulatory myokines can facilitate extracellular vesicle uptake in PCa cells, circulatory extracellular vesicle levels and extracellular vesicle-cargo contents. Finally, the growth of PCa cells will be examined when the cells are exposed to collected extracellular vesicles and serum before and after an acute bout of exercise.

## Methods

### Study design

**Fig. 2:**
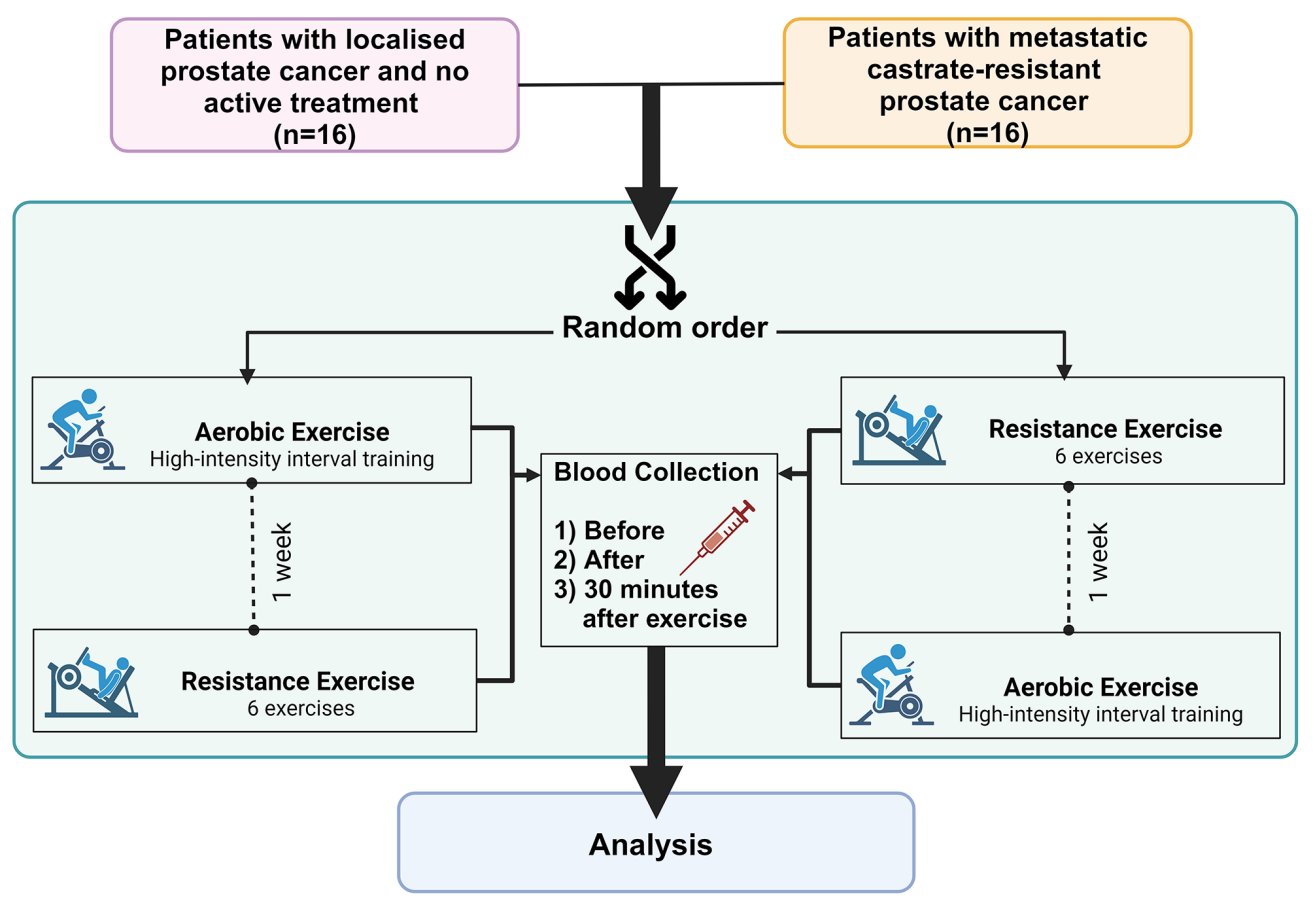
Study design

The proposed study investigates of the effect of an acute bout of exercise with the pre-exercise measurement used as the control condition in a repeated measures design (Fig. 2). Thus, a single-group cross-over design (aerobic and resistance exercise) will be used for two different patient groups (localised and advanced PCa) by capitalising on two of our ongoing trials [21, 22]. Based on our previous investigations [11]of the tumour suppressive role of serum collected after a single bout of high-intensity aerobic exercise in patients with metastatic mCRPC (Cell Index Area Under the Curve (AUC) from Real-Time Cellular Analysis: Pre: 284.78 ± 36.27, Post: 243 ± 35.19, ΔPre-Post: 41.22 ± 34.02), 14 patients will be required to achieve 80% power at an α-level of 0.05 (two-tailed) to detect a 10% difference (AUC 28 units) in total cell growth AUC. As such, a total of 32 patients will be recruited to provide a 10% buffer to any potential sampling and analysis loss – 16 patients under active surveillance and 16 patients with mCRPC.

### Patient recruitment and inclusion/exclusion criteria

Patients from two ongoing trials at the Exercise Medicine Research Institute (EMRI), Edith Cowan University, Western Australia, the Active Surveillance [22] and INTERVAL-GAP4 [21], will be recruited to participate in the study. Detailed inclusion and exclusion criteria are presented in Galvao et al. [22] and Newton et al. [21]. In brief, for Active Surveillance, inclusion criteria were: (1) histologically proven adenocarcinoma of the prostate, (2) no prior therapy for PCa, (3) fit for curative intent therapy, (4) clinical stage ≤ T2, (5) Gleason pattern 4 disease on biopsy, and (6) PSA ≤ 10ng/ml. For the INTERVAL-GAP4 project, patients with mCRPC were recruited. mCRPC was defined as adenocarcinoma of the prostate with progression of systemic metastatic disease despite castrate levels of testosterone (< 50ng/ml) obtained either via orchiectomy or undergoing ADT with a gonadotropin-releasing hormone agonist or antagonist. Exclusion criteria for both trials included patients with acute illness or any musculoskeletal, cardiovascular or neurological disorder that could inhibit exercise performance or put participants at risk from exercising [21, 22]. Recruitment for MYEX will be made among the patients who commenced the interventions and no additional screening is required.

### Ethics

The MYEX trial capitalises on ongoing projects with ethical approval, Active Surveillance [22] (ID: 17,072) and INTERVAL-GAP4 [21] (ID: 13,236). Ethics amendments for both ongoing projects have been approved by the Human Ethics Committee at Edith Cowan University for data and blood sample collection while providing two acute exercise sessions for the participants.

### Exercise protocol and sample collection

**Fig. 3:**
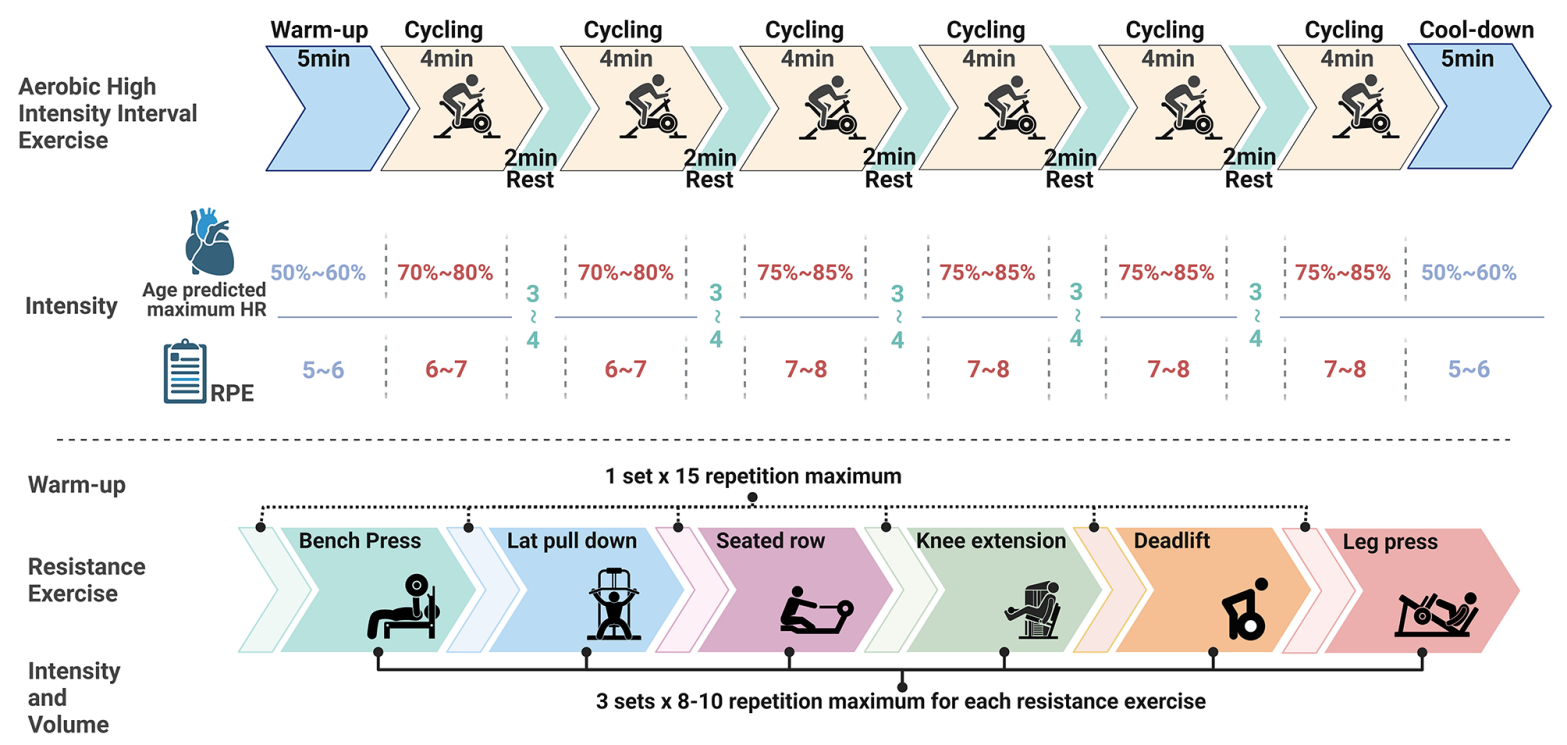
Exercise protocol Aerobic high-intensity interval exercise and resistance exercise will be performed in random order 1 week apart. Aerobic high-intensity interval exercise consists of 6 × 4-minute bouts of cycling at 70 ~ 85% of age-predicted maximum heart rate (RPE 6 ~ 8) with 2 min of low-intensity active recovery (RPE 3 ~ 4) between bouts, preceded by a 5-minute aerobic warm-up and concluding with a 5-minute cool-down. Resistance exercise consists of 6 different exercises involving the major muscle groups (bench press, lat pull down, seated row, knee extension, deadlift, and leg press). For each exercise, 1 set at ~ 15 repetition maximum (RM) will be undertaken as a warm-up, followed by 3 sets at 8–10 RM. Resistance exercise will not be performed to neuromuscular failure, but sets will cease with 1–2 repetitions in reserve

Two single bouts of aerobic or resistance exercise (~ 45 min duration) will be provided to participants in random order one week apart (Fig. 2). On the day of the exercise session, patients will abstain from food and drink (except for water) for 2 h prior to exercise. Aerobic exercise will consist of 5 min warm-up at an intensity of 60–65% of the age-predicted maximum heart rate (estimated as 220-age) or an intensity of 3–4 on the 0–10 Borg rating of perceived exertion (RPE) scale [23]. This will be followed by 6 sets of 4 min of high-intensity cycling at 75–85% of age-predicted maximum heart rate (6–8 RPE) with 2 min active recovery at 3–4 RPE between high-intensity bouts (Fig. 3). The resistance exercise session will consist of 4 sets of 6 exercises involving the major muscle groups (i.e., bench press, lat pull down, seated row, knee extension, deadlift, leg press). The first set of each exercise will be at a load that the participant can lift for approximately 15 repetitions. The subsequent three sets will be at an intensity of 8–10 RM (based on familiarisation loads) with each set completed with 1–2 repetitions in reserve (i.e. not to neuromuscular failure) (Fig. 3).

### Data/Sample collection and analysis

The patient’s medical history, demographic information, and body composition measures (dual-energy x-ray absorptiometry) obtained during patient screening for the two ongoing trials [21, 22] will be shared. In addition, 22 ml of blood will be collected before, immediately after and 30 min after each exercise session by a certified phlebotomist using EDTA-coated vacutainers.

The collected blood will be immediately processed for plasma and isolated for extracellular vesicles. Plasma will be separated immediately by centrifugation (1000 g, 4 °C, 10 min). Subsequently, 4 ml of plasma will be freshly used for extracellular vesicle purification via ultracentrifugation, and the remaining plasma will be frozen at -70 °C. The isolated extracellular vesicles will be tagged with a specific antibody, alpha-sarcoglycan, CD81, and CD45, to characterise skeletal muscle-derived extracellular vesicles and quantified using flow cytometry [20, 24]. The remaining isolated extracellular vesicles will be stored at -70℃ until additional analysis (cell growth analysis and extracellular vesicle content quantification).

Circulating myokines and extracellular vesicle cargo content (e.g., IL-6, IL-15, OSM, SPARC, and fibroblast growth factor [FGF]-21) will be measured using ELISA. The PCa cell lines (LNCaP, DU145, and PC-3) will be cultured by exposing the cells with isolated extracellular vesicles with pre-exercised and post-exercise plasma from the participants to evaluate their effect on cell growth. Finally, the isolated extracellular vesicles will be tagged using alpha-sarcoglycan and stained with fluorescence secondary antibodies, and the uptake of extracellular vesicles will be observed using fluorescence microscopy and image analysis software (Image J, NIH, USA) (detailed protocol is presented in ref [25]).

### Statistical analysis

The normality of outcomes will be tested using the Shapiro-Wilk test and Q-Q plot. Generalised estimating equation (GEE) linear regression models will be used to analyse the difference between outcome measures (circulating SMEV levels, circulating myokines, cell growth [with the presence of extracellular vesicles and extracellular vesicles + plasma], and semi-quantitative outcomes of SMEV uptake) for 3 blood collection time points (pre-, immediately post-, and 30 min post-exercise) for each exercise mode. In addition, two-way analysis of variance (ANOVA) or analysis of covariance (ANCOVA) adjusted for pre-exercise outcome measures will be used as appropriate to detect the differences in outcome measures between exercise types (aerobic exercise, *n* = 32 vs. resistance exercise, *n* = 32). Sub-group analysis will also be performed by dividing the patients into two groups (localised PCa: aerobic, *n* = 16 vs. resistance, *n* = 16; mCRPC: aerobic, *n* = 16 vs. resistance, *n* = 16).

## Patient and public involvement

The Exercise Medicine Research Institute has established a consumer representative group to facilitate research focussed on patient needs. Group members are former and current participants of previous and ongoing trials at EMRI, as well as their carers / partners. The members volunteer their time to provide consumer perspectives on research questions and directions. In addition to direct consumer involvement, local clinicians provided further information on patient priorities. Specifically, the study clinicians (AR, TC) consult within the major public and private hospitals in Perth, Western Australia, each with high PCa caseloads, thus we have used patient priorities, patient experience and patient preference to help inform the development of the research questions and outcome measures.

## Discussion

Over the past two decades, there has been growing evidence supporting the positive impact of physical activity and exercise on cancer outcomes and treatment-related adverse effects for patients with cancer [1]. As such, international and national organisations in exercise and oncology have provided exercise recommendations and guidelines [26–28], which have influenced clinical cancer management. Although these guidelines have been informed by clinical and epidemiological studies [26–28], there remains a need for a greater mechanistic understanding of how exercise affects cancer biology in order to optimize exercise medicine for patients with cancer [6]. Aerobic and resistance exercise induce highly distinct biological responses and adaptations in skeletal muscle and circulatory factors [29]. A priority is therefore to further our understanding of the differential biological impact of these two primary exercise modes [6] in order to enhance cancer management via targeted exercise medicine.

Previously, we have reviewed the tumour suppressive potential of skeletal muscle-induced myokines [6]. Further, the direct suppressive role of exercise-induced systemic content alteration was confirmed by our series of studies investigating the impact of long-term exercise therapy and acute exercise on circulatory myokines and the tumour-suppressive effect of exercise-conditioned serum in patients with PCa [7, 9–11]. Knowledge generated from the MYEX trial will further enhance understanding of the mechanisms by which exercise influences tumour biology and cancer suppression. Specifically, examining the two primary exercise modes in patients with localised or advanced PCa will have an immediate translational impact on the field of exercise oncology.

## Data Availability

No datasets were generated or analysed during the current study.

## Declarations

### Authors’ information (optional)

Not appliable.
